# Cognitive and Functional Decline Among Long-Term Care Residents

**DOI:** 10.1001/jamanetworkopen.2025.5635

**Published:** 2025-04-23

**Authors:** Ramtin Hakimjavadi, Christina Y. Yin, Mary Scott, Robert Talarico, Tim Ramsay, Colleen Webber, Douglas Manuel, Aliza Moledina, Amy T. Hsu, Celeste Fung, Sharon Kaasalainen, Jackie Kierulf, Frank Molnar, Sandy Shamon, Benoît Robert, Paul E. Ronksley, Peter Tanuseputro, Kumanan Wilson, Daniel Kobewka

**Affiliations:** 1 Faculty of Medicine, University of Ottawa, Ottawa, Ontario, Canada; 2 Ottawa Hospital Research Institute, Ottawa, Ontario, Canada; 3 Bruyère Research Institute, Ottawa, Ontario, Canada; 4 Public Health Agency of Canada, Ottawa, Ontario, Canada; 5 ICES, uOttawa site, Ottawa, Ontario, Canada; 6 School of Epidemiology and Public Health, University of Ottawa, Ottawa, Ontario, Canada; 7 Department of Medicine, University of Ottawa, Ottawa, Ontario, Canada; 8 Department of Family Medicine, Faculty of Medicine, University of Ottawa, Ottawa, Ontario, Canada; 9 St Patrick’s Home of Ottawa, Ottawa, Ontario, Canada; 10 School of Nursing, Faculty of Health Sciences, McMaster University, Hamilton, Ontario, Canada; 11 Division of Geriatric Medicine, Department of Medicine, University of Ottawa, Ottawa, Ontario, Canada; 12 Department of Family and Community Medicine, Division of Palliative Care, University of Toronto, Toronto, Ontario, Canada; 13 Perley Health, Ottawa, Ontario, Canada; 14 Department of Community Health Sciences, University of Calgary, Alberta, Canada; 15 Department of Family Medicine and Primary Care, Li Ka Shing Faculty of Medicine, University of Hong Kong, Hong Kong Special Administrative Region, China; 16 Ottawa Hospital Civic Campus, Ottawa, Canada

## Abstract

**Importance:**

Care decisions for long-term care (LTC) residents should be frailty-informed to maximize well-being and avoid burdensome treatments that do not align with patient wishes.

**Objective:**

To investigate the incidence and time spent living with severe impairment among LTC residents to help inform person-centered decision-making.

**Design, Setting, and Participants:**

This retrospective cohort study was conducted among a population-based cohort of incident admissions to LTC facilities between April 1, 2013, and March 31, 2018, determined using administrative health data in Ontario, Canada. Ontario residents aged 65 years or older who were admitted to LTC were included. Participants were followed up until death, discharge, or April 1, 2023. Data analysis was completed from October 17, 2023, to March 31, 2024.

**Main Outcomes and Measures:**

Outcomes were states of impairment that care partners identified as meaningful and some considered worse than death. The incidence of total care dependence, inability to make any decisions, inability to communicate, and incontinence of stool or urine was described. Survival after becoming impaired, characteristics of residents when they became impaired, and characteristics of those who survived for more than 1 year with each impairment were described. Residents at risk of a specific impairment (at-risk residents) were those who did not already have the impairment at admission.

**Results:**

A total of 120 238 residents admitted to LTC (mean [SD] age, 84.3 [7.7] years; 77 868 female [64.8%]) were included. By the end of follow-up, 22 018 of 109 830 at-risk residents (20.0%) had become permanently unable to make decisions, 9138 of 118 132 at-risk residents (7.7%) had become permanently unable to communicate, 15 711 of 116 848 at-risk residents (13.4%) had developed total care dependence, and 30 449 of 92 974 at-risk residents (32.8%) had developed incontinence of stool or urine. Median (IQR) survival time was shortest for residents who entered a state of total care dependence (45 [5-310] days) and longest for those with newly developed incontinence of stool or urine (356 [79-1031] days). Younger residents (eg, median [IQR] survival after developing total care dependence, 133 (17-735) days for ages <80 years vs 30 (4-217) days for ages ≥80 years) and those with dementia at admission (eg, median [IQR] survival after developing the inability to make decisions, 318 [40-1020] days with dementia vs 74 [4-474] days without dementia) had longer median survival after entering a state of severe impairment.

**Conclusions and Relevance:**

In this study, severe permanent impairment in function and cognition were common and often present near the end of life for LTC residents, but a minority of residents lived in these states for years. These results suggest that building shared understanding and open communication about the natural course of frailty trajectories for LTC residents may support resident-centered medical decision-making.

## Key Points

**Question:**

What is the incidence of severe cognitive and functional impairment among people admitted to long-term care (LTC), and how long do they live after becoming severely impaired?

**Findings:**

In this cohort study of 120 238 LTC residents followed up for 5 years, 20.0% lost decision-making capacity, 13.4% lost communication ability, 13.4% became fully dependent, and 32.8% developed incontinence. Median survival was 45 days after becoming fully dependent and 356 days after becoming incontinent.

**Meaning:**

This study found that severe impairment was common after admission to LTC, underscoring the need to understand incidence and what it means for prognosis to support resident-centered decision-making.

## Teaser

This cohort study investigates the incidence of severe cognitive and functional impairment and survival after impairment among long-term care residents in Canada.

## Introduction

Many long-term care (LTC) residents experience cognitive impairments and functional limitations; many have dementia and live at their LTC home until the end of life.^1,2,3^ These residents follow a frailty trajectory with no clear terminal phase; instead, they experience a progressive decline in cognition and physical function after admission due to the accumulation of chronic conditions.^4,5,6,7^ Care for these LTC residents must be guided by their wishes and goals, ensuring dignity and quality of life for residents and care partners.^8^ The standard care practice of providing longevity can leave residents in states that they consider worse than death, contradicting the goals that residents view as most important.^9,10^ In Canada, approximately 71% of LTC residents have do-not-resuscitate (DNR) orders and 26% have do-not-hospitalize (DNH) orders.^11^ Despite these advance care directives, 25% of LTC residents have at least 1 transfer to the hospital every 6 months, and many die shortly after a hospital transfer.^12,13,14^

Patients, including healthy outpatients and those with serious illness, consider states such as being “unable to get out of bed,” “unable to communicate,” or “unable to reason or remember” as worse than death, and yet these outcomes are rarely explicitly discussed.^9,10,15,16^ Pursuing longevity-focused care when a person is living in a state they consider worse than death is contrary to resident-centered principles for which LTC homes strive. However, pursuit of longevity against a patient’s wishes may occur because preferences change when death is imminent and because substitute decision-makers and clinicians are partial toward life-prolonging treatment when clinical outcomes or resident preferences are uncertain.^17^ The US Institute of Medicine Committee on Improving Quality in Long-Term Care reported that states of disability that some individuals may perceive as worse than death have not been described in LTC settings, and the demographic, health, and other characteristics of individuals in these states require further study.^18^ Given the importance of these outcomes to LTC residents and the lack of existing evidence, we sought to describe new severe permanent cognitive and functional impairment among newly admitted LTC residents and describe characteristics of residents who live for a prolonged time with severe impairments.

## Methods

### Study Design and Population

We conducted a retrospective cohort study of LTC residents in Ontario, Canada, using health administrative data housed at ICES (formerly the Institute for Clinical Evaluative Sciences). ICES is an independent nonprofit research institute whose legal status under Ontario’s health information privacy law allows ICES to collect and analyze health care and demographic data without consent for health system evaluation and improvement. The use of the data in this project is authorized under section 45 of Ontario’s Personal Health Information Protection Act and does not require review by a research ethics board. This report adheres to the Strengthening the Reporting of Observational Studies in Epidemiology (STROBE) reporting guideline for cohort studies.

We created an incident cohort of Ontarians aged 65 years or older who were admitted to LTC facilities between April 1, 2013, and March 31, 2018, and followed up with these individuals until death, discharge, or April 1, 2023. A resident’s first admission assessment was considered the index date. We excluded 215 individuals who had a missing birth date or sex information, 9197 individuals older than 105 years or younger than 65 years at the index admission to LTC, and 8936 individuals who had a previous admission from 2010 to 2013. LTC residents who were transferred between facilities (ie, LTC home to LTC home or LTC home to hospital) stayed in the cohort.

### Data Sources

We used the Continuing Care Reporting System (CCRS) and the Ontario Registered Persons Database (RPDB). The CCRS collects sociodemographic and clinical information on all long-stay LTC residents who have received a Resident Assessment Instrument Minimum Data Set (RAI-MDS) 2.0 assessment. Assessments are done at entry to LTC, quarterly (every 92 days or sooner), and with any significant health status change. The RPDB provides information on the vital status of all Ontario residents. These datasets were linked using unique encoded identifiers and analyzed at ICES.

### Variables

Baseline characteristics of LTC residents were obtained from the CCRS. Health-related characteristics were collected from each resident’s first recorded RAI-MDS assessment after admission to LTC and included activities of daily living (ADL)^19^; Cognitive Performance Scale score^20^; Changes in Health, End-Stage Disease, Signs, and Symptoms Scale (CHESS) score (a measure of medical instability and risk of death)^21^; chronic conditions, including dementia, diabetes, and stroke; number of medications; and various social and mood-related behaviors. Detailed descriptions of all variables are available in eTable 1 in Supplement 1.

### Outcomes

We identified 4 outcomes, 2 to describe cognitive impairment and 2 to describe functional impairment. To select our outcomes, we first compiled items and composite scales on quality of life in RAI-MDS. We then consulted with our patient family advisory council with LTC experience to identify resident-important outcomes for decision-making. Based on this meeting, we created a short list of resident-important outcomes and then examined the distribution of each outcome in our cohort, excluding those that were already present for most residents at admission or those that rarely occurred. The research team, including patient partners, clinicians, and researchers (C.Y.Y., C.F., J.K., S.S., B.R., and D.K.), met to finalize the outcome list.

We selected 2 cognitive outcomes: (1) inability to make decisions, defined as a rare or absent ability to make everyday decisions for oneself (eg, when to eat and what to wear) based on Cognitive Performance Scale scores of 5 or 6^22^ and (2) inability to communicate, defined as being unable to understand or be understood by others. We defined 2 functional outcomes: (1) total care dependence, defined as being totally dependent on others for personal hygiene (eg, bathing, grooming, and dental care), toileting, moving, and eating based on an ADL self-performance hierarchy score of 6^19^ and (2) incontinence of stool or urine, defined as requiring an adult diaper. We ensured that cognitive and functional states were permanent, meaning that residents remained in the state of impairment with no improvement until the end of follow-up or death. States were not mutually exclusive. All cognitive and functional outcomes were defined using variables from RAI-MDS 2.0 assessments.

### Statistical Analysis

We conducted a series of descriptive analyses presenting characteristics of residents entering the state of impairment using means with SDs and medians with IQRs for continuous variables and frequencies and proportions for categorical variables. We described the cumulative incidence of impairments while considering death as a competing event. Residents who died were considered to be no longer at risk of developing the impairment, avoiding overestimation of the risk of impairment. To explore characteristics of individuals who lived for a prolonged time with severe impairment, we compared residents who survived for 1 year or more after entering these states of impairment with those who died within the first year using absolute standardized differences. We also described the proportion who survived for more than 1 year with severe disability by presence or absence of DNH orders, DNR orders, and both DNR and DNH orders. We used the Kaplan-Meier method to present survival data for residents after they entered each state of impairment and then stratified by a diagnosis of dementia or age 80 years or older on admission to determine whether younger age or absence of dementia were associated with prolonged survival time with impairment. Age 80 years or older was selected because it aligns with the oldest-old in Canada.^23^ We accounted for the potential difference in survival by categorizing on survival time and conducting stratified analysis by age and dementia status. Our approach described resident characteristics and how survival varied among residents, which did not involve adjusted analyses. All analyses were conducted using SAS statistical software version 9.4 (SAS Institute). Data analysis was completed from October 17, 2023, to March 31, 2024.

## Results

Our cohort included 120 238 residents admitted to 650 LTC facilities between April 1, 2013, and March 31, 2018 (mean [SD] age, 84.3 [7.7] years; 77 868 female [64.8%]) (Table). More than half of residents had a diagnosis of dementia at baseline (69 795 residents [58.0%]). The median (IQR) survival was 903 (329-1772) days. At admission, 10 408 residents (8.7%) had severe cognitive impairment, leaving 109 830 residents at risk of developing severe cognitive impairment; 2106 residents (1.8%) had severe communication deficits, leaving 118 132 residents at risk of developing this impairment; 3390 residents (2.8%) were totally dependent on others for ADL, leaving 116 848 residents at risk of developing this impairment; and 27 264 residents (22.7%) had bowel or bladder incontinence, leaving 92 974 residents at risk of developing bowel or bladder incontinence (Table).

**Table.** Baseline Sociodemographic and Clinical Characteristics

Characteristic^a^	Residents, No. (%) (N = 120 238)
Age at admission, mean (SD), y	84.3 (7.7)
Sex	
Female	77 868 (64.8)
Male	42 370 (35.2)
Highest level of education completed	
≤Grade 11	30 899 (25.7)
High school	19 199 (16.0)
Technical or trade school	5159 (4.3)
Some college or university	16 220 (13.6)
Unknown	47 430 (39.4)
CPS score	
0 = Intact	18 021 (15.0)
1 = Borderline intact	13 666 (11.4)
2 = Mild impairment	27 912 (23.2)
3 = Moderate impairment	41 304 (34.4)
4 = Moderate-severe impairment	8925 (7.4)
5 = Severe impairment	8010 (6.7)
6 = Very severe impairment	2398 (2.0)
ADL Self-Performance Hierarchy Scale score	
0 = Independent	3645 (3.0)
1 = Supervision	7779 (6.5)
2 = Limited	20 599 (17.1)
3 = Extensive	36 601 (30.4)
4 = Maximal	28 284 (23.5)
5 = Dependent	19 938 (16.6)
6 = Total dependence	3390 (2.8)
CHESS score	
0 = No health instability	56 100 (46.7)
1 = Minimal health instability	41 392 (34.4)
2 = Low health instability	16 779 (14.0)
3 = Moderate health instability	4599 (3.8)
4 = High health instability	1157 (1.0)
5 = Very high health instability	209 (0.2)
Chronic conditions	
Dementia	69 795 (58.0)
Stroke	21 962 (18.3)
Diabetes	32 140 (26.7)
Parkinson disease	7690 (6.4)
Multiple sclerosis	535 (0.4)
Cancer	12 372 (10.3)
Congestive heart failure	17 314 (14.4)
Hypertension	79 357 (66.1)
Stroke	21 967 (18.3)
Kidney failure	12 913 (10.7)
Arteriosclerotic heart disease	17 518 (14.6)
Emphysema or COPD	19 262 (16.0)
Liver disease	1262 (1.1)
Mental health disorders	
Depression	25 963 (21.6)
Anxiety	10 933 (9.1)
No. of medications, mean (SD)	9.99 (4.45)

Abbreviations: ADL, activities of daily living; CHESS, Changes in Health, End-Stage Disease, Signs, and Symptoms Scale; CPS, Cognitive Performance Scale; COPD, chronic obstructive pulmonary disease.

^a^ Characteristics are given for the cohort of residents aged 65 years or older at admission to long-term care, 2013 to 2018.

### Cumulative Incidence of Cognitive or Functional Impairment

**Figure 1.**
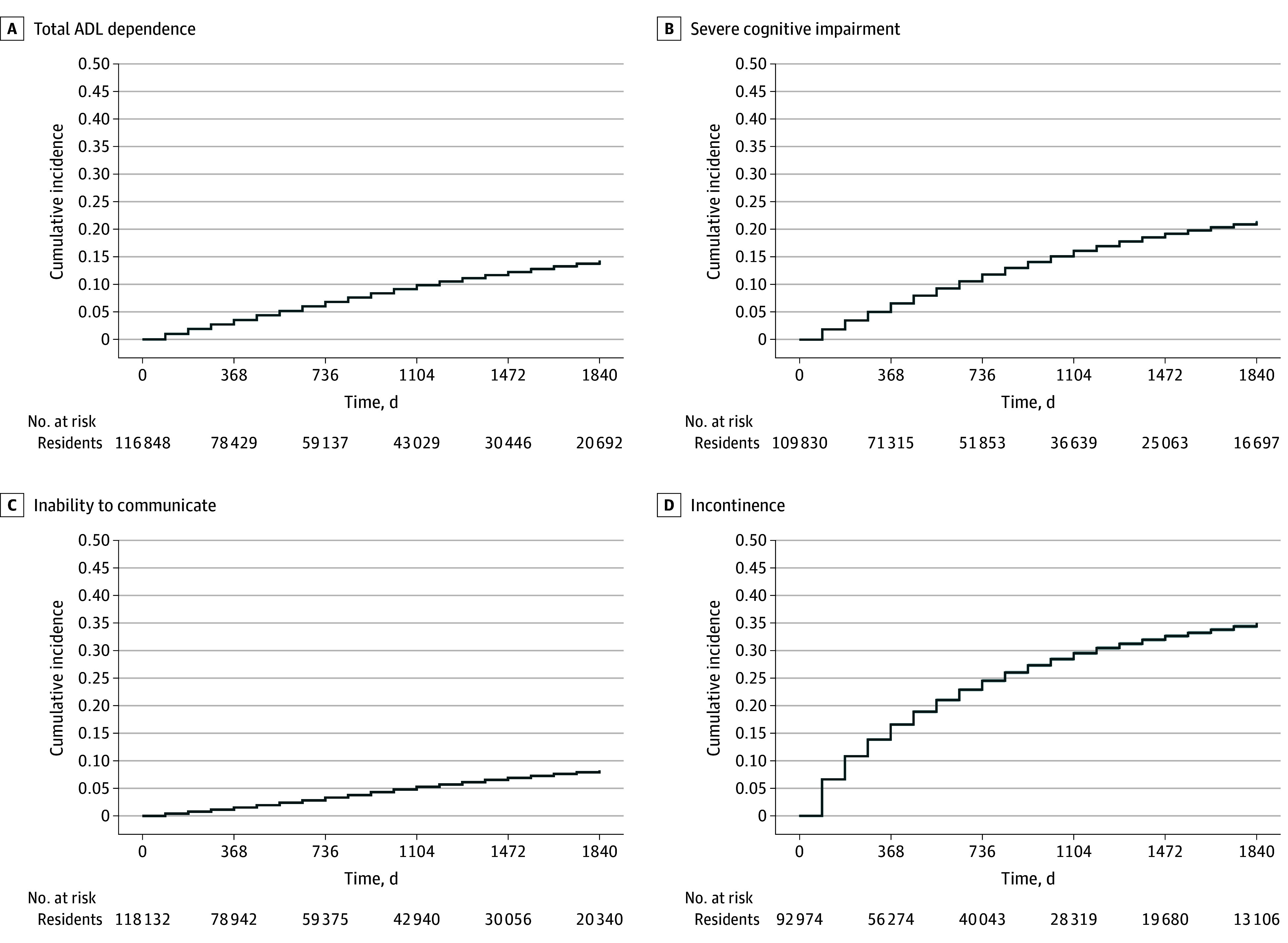
Cumulative Incidence of Entering a State of Impairment Over 5-Year Follow-Up Cumulative incidence curves are shown stratified by entering a state of total ADL dependence: being unable to perform any ADL independently (A); severe cognitive impairment: being unable to make any decisions for self (B); inability to communicate: being unable to understand others or make self understood (C); and incontinence: being incontinent of urine or stool requiring an adult diaper (D).

We assessed the development of each impairment among residents who did not already have the impairment at admission (at-risk residents). By the end of follow-up, 22 018 of 109 830 at-risk residents (20.0%) had developed a permanent inability to make decisions, 9138 of 118 132 at-risk residents (7.7%) had developed permanent inability to communicate, 15 711 of 116 848 at-risk residents (13.4%) had developed total care dependence, and 30 449 of 92 974 at-risk residents (32.8%) had developed incontinence of stool or urine (Figure 1).

### Survival After Entering a State of Impairment

After developing impairment, 9322 residents (42.3%), 3564 residents (39.0%), 3330 residents (21.2%), and 14 750 residents (48.4%) lived for more than 1 year being unable to make decisions, unable to communicate, totally dependent for care, or with incontinence, respectively. Fewer residents with DNR and DNH orders lived for more than 1 year after entering states of permanent impairment. After developing impairment, 2222 of 5715 residents (38.9%), 877 of 2336 residents (37.5%), 737 of 3862 residents (19.1%), and 3371 of 7592 residents (44.4%) who had DNR and DNH orders lived for more than 1 year being unable to make decisions, unable to communicate, totally dependent for care, or with incontinence, respectively (eTable 2 in Supplement 1). The trend of shorter survival with severe impairment was similar for residents with only a DNR or DNH order (eTable 2 in Supplement 1).

Residents who lived for more than 1 year with severe cognitive impairment were younger (mean [SD] age, 84.78 [7.39] years vs 87.41 [7.03] years), more likely to be female (6740 female [72.3%] vs 8121 female [64.0%]), and more medically stable as indicated by the CHESS score (eg, 2315 residents [24.8%] vs 1121 residents [8.8%] with no health instability [score of 0]) and had a lower prevalence of stroke (1511 residents [16.2%] vs 2801 residents [22.1%]) and diabetes (1982 residents [21.3%] vs 3232 residents [25.5%]) compared with those who died within 1 year. We observed similar differences between residents who survived for more than 1 year compared with those who survived less than 1 year with the inability to communicate, total care dependence, and incontinence of stool or urine (eTable 2 in Supplement 1).

The median (IQR) survival time was shortest for residents who had developed total care dependence (45 [5-310] days), followed by those who developed the inability to communicate (205 [9-963] days), inability to make decisions (262 [23-924] days), and incontinence of stool or urine (356 [79-1031] days) (eFigure in Supplement 1). In the complete cohort, 41 698 of 120 238 residents (65.2%) had a DNR order and 30 587 of 120 238 residents (25.4%) had a DNH order. Residents with DNR and DNH orders did not live as long after becoming impaired as those who did not have a DNR and DNH order. Median (IQR) survival for residents with DNR and DNH compared with those without DNR and DNH was 202 (14-765) days vs 326 (37-1164) days after becoming unable to make decisions, 179 (7-889) days vs 255 (17-1373) days after becoming unable to communicate, 28 (3-239) days vs 63 (8-425) days after becoming totally dependent for care, and 294 (62-871) days vs 475 (107-1326) days after becoming incontinent .

**Figure 2.**
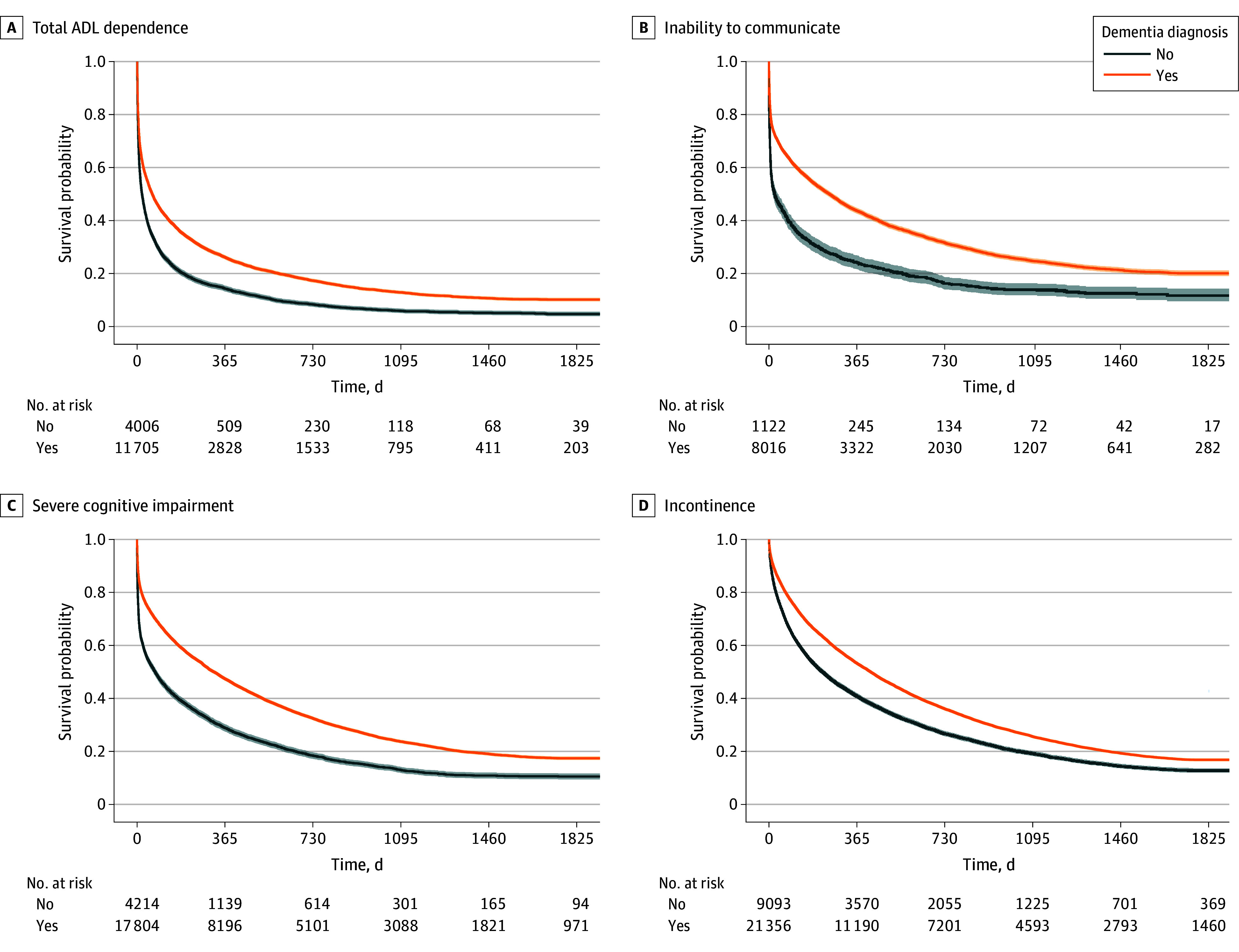
Survival Over 5-Year Follow-Up After Entering a State of Impairment by Dementia Status Kaplan-Meier survival curves for death are shown stratified by entering a state of total ADL dependence: being unable to perform any ADL independently (A); inability to communicate: being unable to understand others or make self understood (B); severe cognitive impairment: being unable to make any decisions for self (C); and incontinence: being incontinent of urine or stool requiring an adult diaper (D) and by dementia status upon long-term care admission. Shaded areas indicate 95% CIs. ADL indicates activities of daily living.

**Figure 3.**
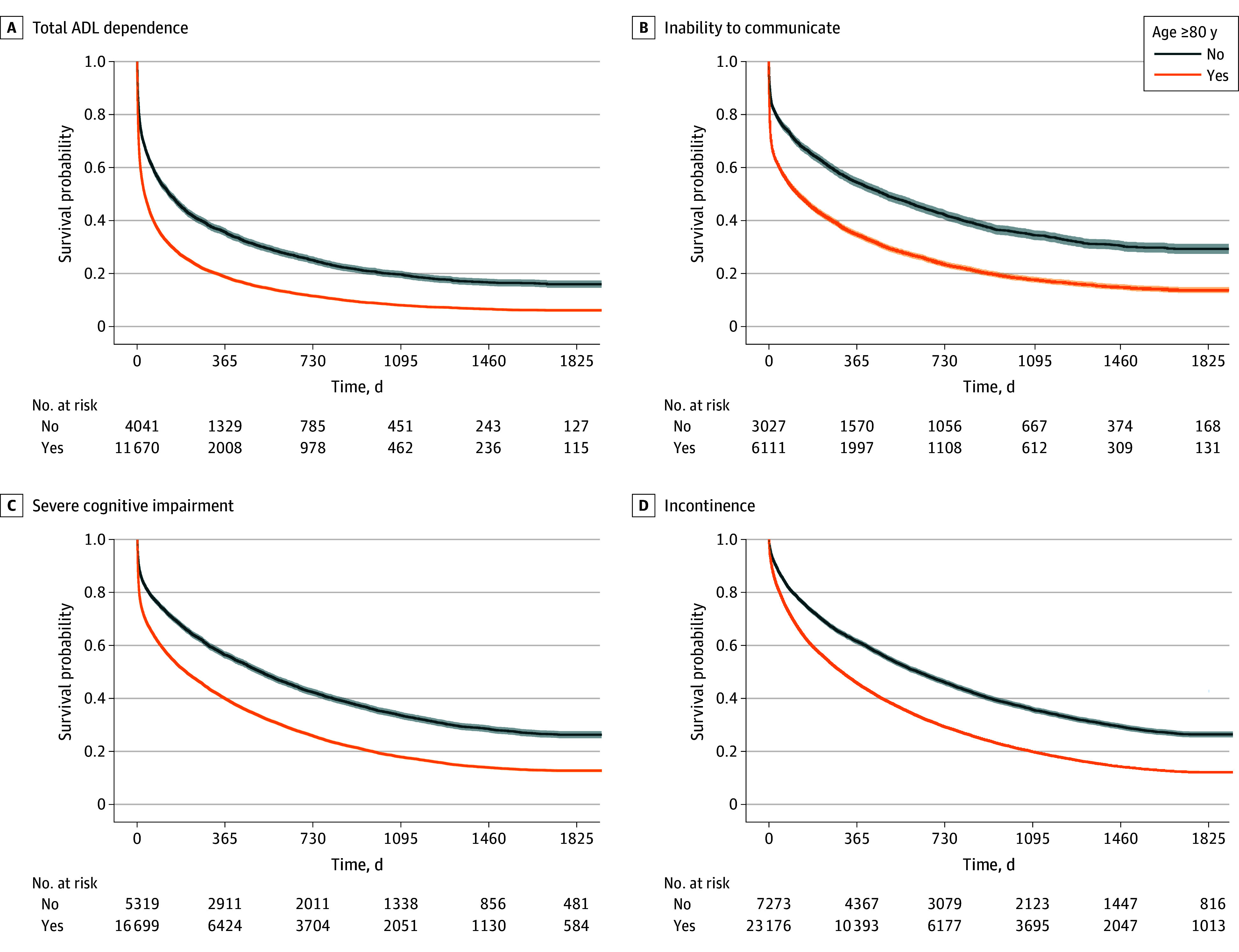
Survival Over 5-Year Follow-Up After Entering a State of Impairment by Age Kaplan-Meier survival curves for death are shown stratified by entering a state of total ADL dependence: being unable to perform any ADL independently (A); inability to communicate: being unable to understand others or make self understood (B); severe cognitive impairment: being unable to make any decisions for self (C); and incontinence: being incontinent of urine or stool requiring an adult diaper (D) and by age (≥80 years vs <80 years) upon long-term care admission. Shaded areas indicate 95% CIs. ADL indicates activities of daily living.

Younger residents and residents with dementia at admission were more likely to live for a prolonged period after they entered a state of severe impairment (Figure 2). Residents who were aged 80 years or older when admitted to LTC had shorter median survival times across each outcome (Figure 3). For example, the median (IQR) survival time after developing total care dependence was 30 (4-217) days for residents aged 80 years or older and 133 (17-735) days for those younger than 80 years. The opposite was observed for residents living with dementia. The median (IQR) survival after developing the inability to make decisions was 318 (40-1020) days for residents with dementia vs 74 (4-474) days for those without dementia. Similarly, the median (IQR) survival after developing the inability to communicate was 250 (14-1078) days for residents with dementia vs 23 (2-336) days for those without dementia.

## Discussion

In this cohort study, we defined states of severe cognitive and functional impairment that are important to residents and family members and described the incidence and survival time with such impairments in a population-level admission cohort of LTC residents. We found that severe cognitive or functional impairment that some individuals may consider worse than death is common after admission to LTC. Mortality was high after residents entered any state of cognitive or functional impairment, particularly among residents with total care dependence. Despite the high mortality, many residents continued to live for prolonged periods with severe impairment; this was especially true among younger residents and those with dementia.

Our outcomes were objectively measurable states of severe impairment that were selected to be resident centered. Residents have reported in interviews that loss of independence and function are more distressing than the thought of impending death.^24^ Our findings highlight the prolonged time spent by some residents in states of severe cognitive or functional impairment. Failing to recognize that a person may consider some states of impairment worse than death is not resident centered or evidence-based.^15^ Older adults with life-limiting illness often prioritize quality of life and prefer end-of-life care that allows for a peaceful death.^25,26^ In a treatment preference study by Fried et al^27^ that surveyed older adults with life-limiting illness, 74.4% of patients declined life-prolonging treatment if it could also cause functional impairment and 88.8% declined treatment if it would also lead to severe cognitive impairment. Knowledge that one may live in a state that is perceived as worse than death can provide clarity and empowerment for shared and well-informed decision-making about when prolonging life is or is not one of the goals of care.

We observed that the time spent living with impairment depended on the type of impairment and that, consistent with other studies, new severe functional impairment heralded a steeper decline approaching death.^4^ Residents who developed total care dependence had a median survival of 45 days, much less than the median survival time for the full cohort (2.5 years [903 days]) or for the other states of impairment measured. This finding is consistent with existing literature on prognostic models for mortality in older adults,^28,29,30^ emphasizing the importance of understanding and incorporating functional states when discussing end-of-life care with residents and care partners.

Residents living with dementia may be at risk of spending more time living in states of impairment than residents who have no cognitive impairment. The increased survival of residents with dementia may reflect the natural history of dementia as a progressive terminal disease with great variability in survival time, ranging from 3 to 15 years.^13,31,32,33,34^ However, the exact reason for the longer survival time after entering states of impairment for residents with dementia compared with those without dementia could not be answered in our study because of the descriptive nature of our analyses. Dementia and cognitive dysfunction have been identified as independent predictors of admission to LTC among community-dwelling older adults,^35,36^ which may be expedited for residents with poor cognition or behavioral and psychological symptoms of dementia.^37^ While earlier admission and longer LTC stay may present more opportunities for care planning and discussion, previous studies have reported uncertainty surrounding when longevity should no longer be a goal for care or what potentially burdensome treatments are acceptable in pursuit of more time alive in persons living with dementia.^38,39,40,41^

DNH and DNR orders usually reflect the resident’s wish to not receive aggressive treatment toward the end of life and indicate an understanding of their declining health trajectory. In our population, 65.2% of residents had DNR orders and 25.4% had DNH orders. DNR and DNH orders were associated with reduced time living with permanent impairments. Our findings suggest that advance directives may play an important role in reducing time spent living with severe disability. Discussing goals for care, including DNR and DNH, with residents and their care partners has the potential to support resident-centered end-of-life decisions.

### Limitations

Our study has several limitations. First, all studies that use administrative data have a risk of bias from misclassification or incomplete capture of variables. To address this limitation, we used validated definitions when possible and maintained consistency with previous research. Second, we did not further stratify by the type of dementia in our analyses (eg, Alzheimer, Lewy body, or frontotemporal dementia) given the available data. Although different dementia subtypes carry distinct trajectories and prognoses, we expect that our findings reflect residents living with Alzheimer dementia given its prevalence relative to other subtypes.^42^ Third, the length of stay observed in this study is subject to local admission and aging-in-place policies, which vary by jurisdiction.^13^ Fourth, while the study aimed to inform resident-centered decision-making in LTC through the identification of resident-important cognitive and functional impairments, resident and caregiver perspectives may differ from those of the patient partners who informed this study. Rather, we provide a foundation for communication and future studies to consider functional and cognitive impairments as outcomes for prognostication. Fifth, our study has limitations in generalizability. Our findings are generalizable to regions with a similar LTC population of mostly older adults with frailty and chronic conditions requiring continuous care. However, our population may be older and have higher levels of frailty and health burdens at admission because of the prioritization of home care in Canada compared with regions with less emphasis on home care or with more flexible LTC eligibility criteria. Additionally, the publicly funded single-payer system in Canada may result in a more homogenous population compared with regions using a third-payer system, which may have a more diverse LTC population. This difference may limit the generalizability of our results to other regions.

## Conclusions

This cohort study found that the incidence of developing severe permanent cognitive or functional impairment was high in the first 5 years of LTC admission. Residents who were younger or living with dementia at admission were more likely to live for a prolonged period in a state of severe impairment. These findings may support clinicians when discussing illness understanding, disease trajectory, and medical decision-making with residents and their families. Discussions about the risk of entering a prolonged state of impairment that some individuals may consider worse than death could provide clarity in helping residents and families make resident-centered decisions about when prolonging life is no longer a goal for medical care.

## Notes

Supplement 1. **eTable 1.** Details on study variables **eTable 2.** Comparison of characteristics at the time of impairment development between residents with shortened (1-year) or prolonged (>1-year) survival after entering a state of cognitive or functional impairment **eFigure.** Survival over the 5-year follow-up after entering a state of impairment

Supplement 2.
Data Sharing Statement
