# Supplementary material for: Cognitive and Functional Decline Among Long-Term Care Residents
Source: JAMA Netw Open. 2025 Apr 23;8(4):e255635. doi: 10.1001/jamanetworkopen.2025.5635 (PMC12019527; doi:10.1001/jamanetworkopen.2025.5635)
Supplement: Supplement 1. — eTable 1. Details on study variables eTable 2. Comparison of characteristics at the time of impairment development between residents with shortened (1-year) or prolonged (>1-year) survival after entering a state of cognitive or functional impairment eFigure. Survival over the 5-year follow-up after entering a state of impairment [file jamanetwopen-e255635-s001.pdf]

Supplemental Online Content

Hakimjavadi R, Yin CY, Scott M, et al. Cognitive and functional decline among long-term care residents. *JAMA Netw Open*. 2025;8(4):e255635. doi:10.1001/jamanetworkopen.2025.5635

eTable 1. Details on study variables

eTable 2. Comparison of characteristics at the time of impairment development between residents with shortened (1-year) or prolonged (>1-year) survival after entering a state of cognitive or functional impairment

eFigure. Survival over the 5-year follow-up after entering a state of impairment

This supplemental material has been provided by the authors to give readers additional information about their work.

© 2025 Hakimjavadi R et al. *JAMA Network Open*.

eTable 1. Details on Study Variables.

**Variable**	**Definition**
ADL Independence	Based on individuals' performance with locomotion, eating, toileting, and personal hygiene. Coded on a 7-point scale, from 0 (independent), 1 (requires supervision), 2 (requires limited assistance with at least 1 of the 4 ADL), 3 (requires at least extensive assistance in personal hygiene or toileting), 4 (requires extensive assistance with eating or locomotion), 5 (total dependence in eating and locomotion), to 6 (total dependence).
CHESS score	Based on 9 assessment items: decline in cognition, decline in ADL, dehydration, edema, shortness of breath, vomiting, end-stage disease, weight loss of 5% or more in the last 30 days or 10% or more in the last 180 days, and leaving 25% or more of food uneaten at most meals.
CPS score	A scale ranging from 0 to 6, with higher scores indicating greater cognitive impairment. The scores correspond to different levels of cognitive function. A score

© 2025 Hakimjavadi R et al. *JAMA Network Open*.

	of 0 indicates intact cognition, a score of 1 signifies borderline intact cognition. Mild impairment is represented by a score of 2, followed by moderate impairment at 3. Scores of 4 and 5 denote moderate to severe and severe impairment, respectively. The highest score, 6, indicates very severe impairment or a comatose state.
ADL, activities of daily living; CHESS, Changes in Health, End-Stage Disease, Signs, and Symptoms Scale; CPS	

© 2025 Hakimjavadi R et al. *JAMA Network Open*.

eTable 2. Comparison of characteristics at the time of impairment development between residents with shortened (1 year) or prolonged (>1 year) survival after entering a state of cognitive or functional impairment.

	a) Severe cognitive impairment			b) Severe communication deficits			c) Total dependence in ADLs			d) Bowel or bladder incontinence		
**Characteristic***	**Survival ≤1 year** n (%)	**Survival >1 year** n (%)	**St. Diff.**	**Survival ≤1 year** n (%)	**Survival >1 year** n (%)	**St. Diff.**	**Survival ≤1 year** n (%)	**Survival >1 year** n (%)	**St. Diff.**	**Survival ≤1 year** n (%)	**Survival >1 year** n (%)	**St. Diff.**
	**N=12,696**	**N=9,322**		**N=5,574**	**N=3,564**		**N=12,381**	**N=3,330**		**N=15,699**	**N=14,750**	
Age	87.41 ± 7.03	84.78 ± 7.39	0.37	86.14 ± 7.26	83.03 ± 7.24	0.43	87.06 ± 7.28	83.82 ± 7.48	0.44	87.24 ± 7.21	84.74 ± 7.47	0.34
Sex, Female	8,121 (64.0%)	6,740 (72.3%)	0.18	3,548 (63.7%)	2,612 (73.3%)	0.21	7,889 (63.7%)	2,354 (70.7%)	0.15	9,967 (63.5%)	10,305 (69.9%)	0.14
Sex, Male	4,575 (36.0%)	2,582 (27.7%)	0.18	2,026 (36.3%)	952 (26.7%)	0.21	4,492 (36.3%)	976 (29.3%)	0.15	5,732 (36.5%)	4,445 (30.1%)	0.14
CHESS score												
0 = No health instability	1,121 (8.8%)	2,315 (24.8%)	0.44	693 (12.4%)	1,362 (38.2%)	0.62	867 (7.0%)	928 (27.9%)	0.57	2,270 (14.5%)	4,624 (31.3%)	0.41
1 = Minimal health instability	1,888 (14.9%)	2,777 (29.8%)	0.36	929 (16.7%)	1,154 (32.4%)	0.37	1,674 (13.5%)	1,156 (34.7%)	0.51	3,677 (23.4%)	5,100 (34.6%)	0.25

© 2025 Hakimjavadi R et al. *JAMA Network Open*.

2 = Low health instability	2,082 (16.4%)	2,351 (25.2%)	0.22	827 (14.8%)	671 (18.8%)	0.11	2,029 (16.4%)	744 (22.3%)	0.15	3,482 (22.2%)	3,102 (21.0%)	0.03
3 = Moderate health instability	2,160 (17.0%)	1,255 (13.5%)	0.1	859 (15.4%)	257 (7.2%)	0.26	2,363 (19.1%)	331 (9.9%)	0.26	2,731 (17.4%)	1,415 (9.6%)	0.23
4 = High health instability	2,801 (22.1%)	519 (5.6%)	0.49	1,098 (19.7%)	97 (2.7%)	0.56	3,053 (24.7%)	133 (4.0%)	0.62	2,170 (13.8%)	458 (3.1%)	0.39
5 = Very high health instability	2,644 (20.8%)	105 (1.1%)	0.66	1,168 (21.0%)	23 (0.6%)	0.69	2,395 (19.3%)	38 (1.1%)	0.63	1,368 (8.7%)	50 (0.3%)	0.41
Chronic conditions												
Dementia	9,720 (76.6%)	7,411 (79.5%)	0.07	4,531 (81.3%)	2,777 (77.9%)	0.08	9,046 (73.1%)	2,530 (76.0%)	0.07	10,075 (64.2%)	10,281 (69.7%)	0.12
Stroke	2,801 (22.1%)	1,511 (16.2%)	0.15	1,159 (20.8%)	488 (13.7%)	0.19	2,832 (22.9%)	622 (18.7%)	0.1	3,094 (19.7%)	2,563 (17.4%)	0.06
Diabetes	3,232 (25.5%)	1,982 (21.3%)	0.1	1,283 (23.0%)	670 (18.8%)	0.1	3,181 (25.7%)	682 (20.5%)	0.12	4,230 (26.9%)	3,600 (24.4%)	0.06
Parkinson's disease	637 (5.0%)	215 (2.3%)	0.14	363 (6.5%)	67 (1.9%)	0.23	755 (6.1%)	134 (4.0%)	0.09	578 (3.7%)	400 (2.7%)	0.06

© 2025 Hakimjavadi R et al. *JAMA Network Open*.

Multiple sclerosis	26 (0.2%)	14 (0.2%)	0.01	5-10 §	<=5 §	0.01	29 (0.2%)	16 (0.5%)	0.04	36 (0.2%)	50 (0.3%)	0.02
Mental health												
Depression	3,746 (29.5%)	2,899 (31.1%)	0.03	1,636 (29.4%)	1,084 (30.4%)	0.02	3,577 (28.9%)	1,033 (31.0%)	0.05	4,250 (27.1%)	4,342 (29.4%)	0.05
Anxiety	885 (7.0%)	452 (4.8%)	0.09	419 (7.5%)	147 (4.1%)	0.15	891 (7.2%)	142 (4.3%)	0.13	929 (5.9%)	690 (4.7%)	0.06
Number of medications (mean ± SD)	9.89 ± 4.78	9.25 ± 3.91	0.15	8.95 ± 4.50	8.38 ± 3.46	0.14	9.57 ± 5.06	9.15 ± 3.97	0.09	10.91 ± 4.79	10.04 ± 4.16	0.19
Advance care directives												
DNR	8,843 (69.7%)	6,044 (64.8%)	0.1	3,697 (66.3%)	2,248 (63.1%)	0.07	8,356 (67.5%)	2,019 (60.6%)	0.14	11,002 (70.1%)	9,204 (62.4%)	0.16
DNH	3,572 (28.1%)	2,286 (24.5%)	0.08	1,489 (26.7%)	897 (25.2%)	0.04	3,212 (25.9%)	757 (22.7%)	0.07	4,320 (27.5%)	3,462 (23.5%)	0.09
DNR and DNH	3,493 (27.5%)	2,222 (23.8%)	0.08	1,459 (26.2%)	877 (24.6%)	0.04	3,125 (25.2%)	737 (22.1%)	0.07	4,221 (26.9%)	3,371 (22.9%)	0.09

CHESS, Changes in Health, End-Stage Disease, Signs, and Symptoms Scale; DNR, Do not resuscitate; DNH, Do not hospitalize; SD, Standard deviation; St. Diff., Absolute Standardized Difference.

*Data were collected from the assessment in the follow-up for which the outcome first occurred, and survival is measured from outcome occurrence.

§ Number suppressed to protect against reidentification risk

© 2025 Hakimjavadi R et al. *JAMA Network Open*.

eFigure 1. Survival over the 5-year follow-up after entering a state of impairment. Note: Kaplan–Meier survival curves for death, stratified by entering a state of inability to make decisions (top-left), inability to communicate (top-right), total care dependence (bottom-left), and incontinence of stool or urine (bottom-right).

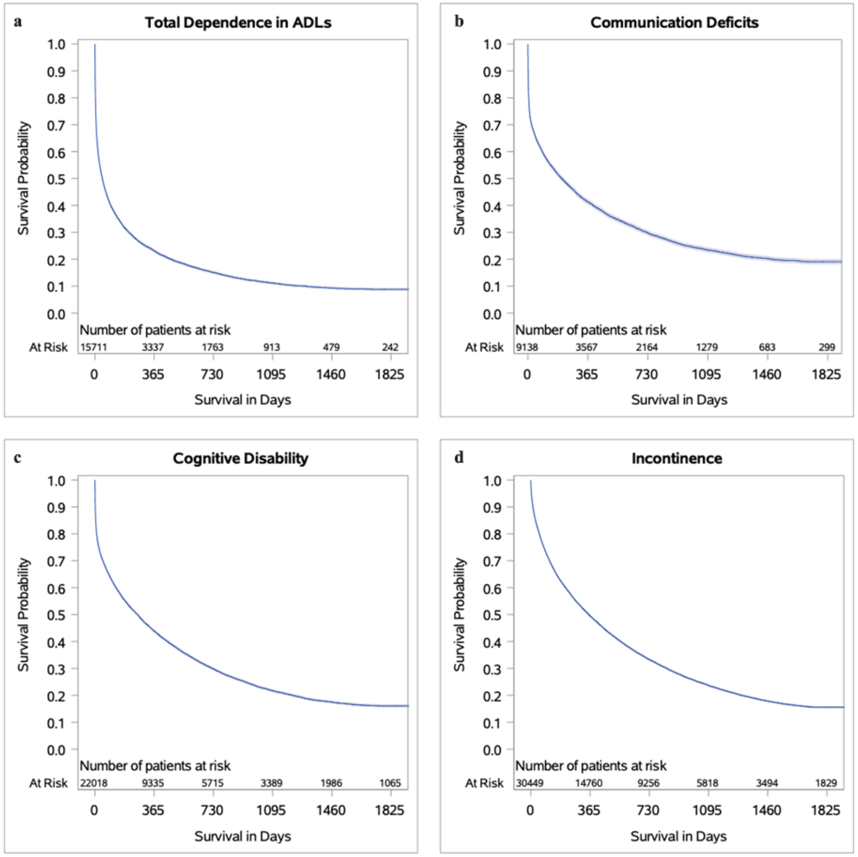

© 2025 Hakimjavadi R et al. *JAMA Network Open*.
