# Supplementary material for: Cognitive and Functional Decline Among Long-Term Care Residents
Source: JAMA Netw Open. 2025 Apr 23;8(4):e255635. doi: 10.1001/jamanetworkopen.2025.5635 (PMC12019527; doi:10.1001/jamanetworkopen.2025.5635)
Supplement: Supplement 2. — Data Sharing Statement [file jamanetwopen-e255635-s002.pdf]

Data Sharing Statement

Hakimjavadi. Cognitive and Functional Decline Among Long-Term Care Residents. *JAMA Netw Open*. Published online April 17, 2025. doi:10.1001/jamanetworkopen.2025.5635

Data
Data available: No

Additional Information
Explanation for why data not available: The dataset is protected under privacy law in my region and can not be shared.
